# Supplementary material for: The interactome of intact mitochondria by cross-linking mass spectrometry provides evidence for coexisting respiratory supercomplexes
Source: Mol Cell Proteomics. 2017 Dec 8;17(2):216–32. doi: 10.1074/mcp.RA117.000470 (PMC5795388; doi:10.1074/mcp.RA117.000470)
Supplement: Supplemental Data [file supp_RA117.000470_133922_0_supp_23978_fzffwf.zip › spectra_annotation/mito_DR_spectra_annotation/148-1-4-1-15-1.pdf]

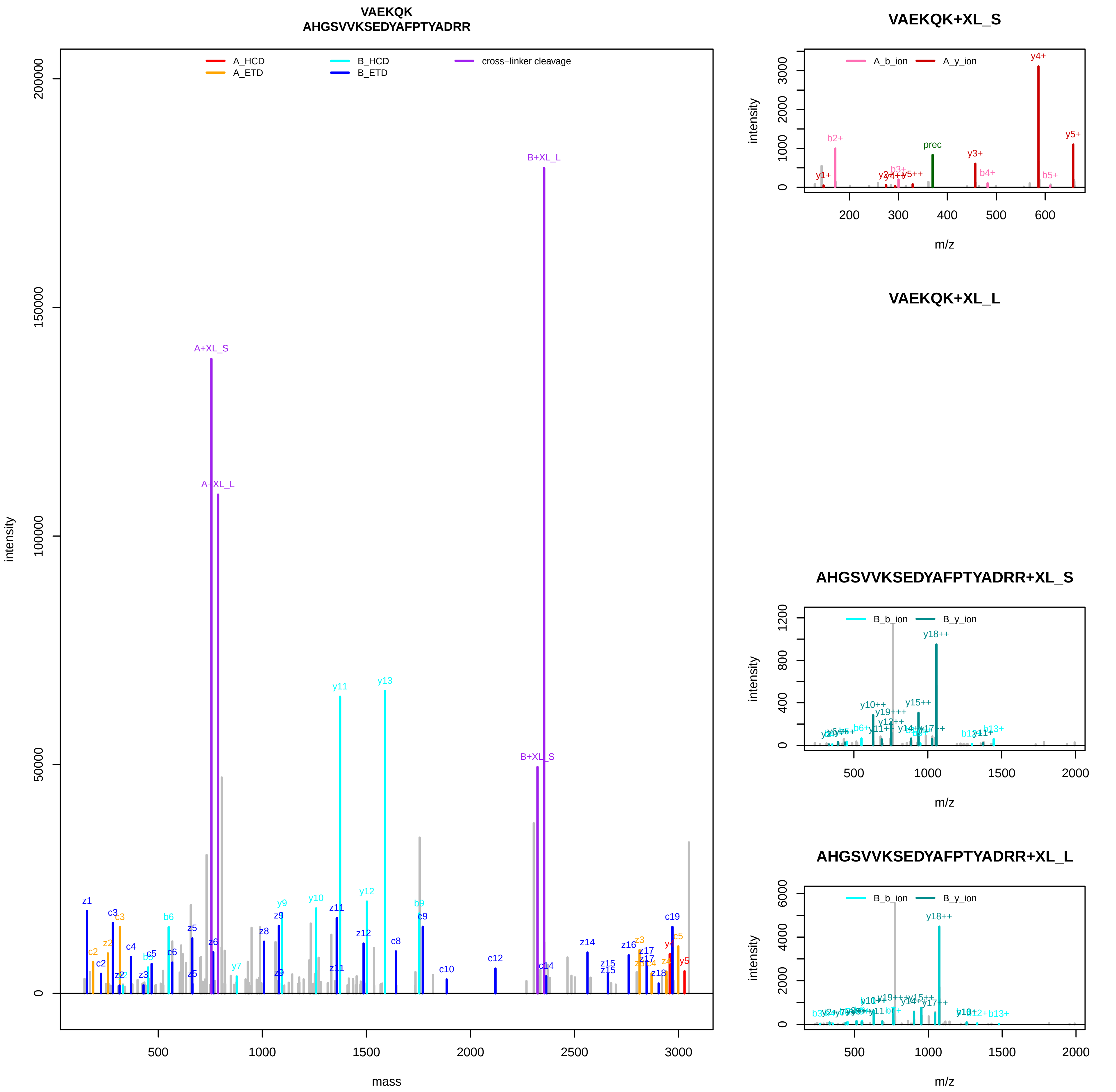
VAEKQK
AHGSVVKSEDYAFPTYADRR
A_HCD
A_ETD
B_HCD
B_ETD
cross−linker cleavage
B+XL_L
A+XL_S
A+XL_L
B+XL_S
y11
y13
y12
y10
y9
z9
z11
z12
c8
c9
b9
c10
c12
c14
z14
z15
z16
z17
z18
c19
c5
y5
z1
c3
c2
z2
c4
c5
b6
c6
z5
z6
z8
y7
intensity
mass
VAEKQK+XL_S
A_b_ion
A_y_ion
y4+
y5+
b2+
prec
y3+
b4+
b5+
y1+
m/z
VAEKQK+XL_L
AHGSVVKSEDYAFPTYADRR+XL_S
B_b_ion
B_y_ion
y18++
y15++
y10++
y19+++
b13+
m/z
AHGSVVKSEDYAFPTYADRR+XL_L
B_b_ion
B_y_ion
y18++
m/z
